# The effect of propolis supplementation on inflammatory factors and oxidative status in women with rheumatoid arthritis: Design and research protocol of a double-blind, randomized controlled

**DOI:** 10.1016/j.conctc.2021.100807

**Published:** 2021-06-23

**Authors:** Elyas Nattagh-Eshtivani, Mohammadhassan Jokar, Hamed Tabesh, Mohsen Nematy, Mohammad Safarian, Naseh Pahlavani, Mona Maddahi, Maryam Khosravi

**Affiliations:** a Department of Nutrition, Faculty of Medicine, Mashhad University of Medical Sciences, Mashhad. Iran; b Rheumatic Diseases Research Center, School of Medicine, Mashhad University of Medical Sciences, Mashhad, Iran; c Department of Medical Informatics, Faculty of Medicine, Mashhad University of Medical Sciences, Mashhad, Iran; d Metabolic Syndrome Research Center, Mashhad University of Medical Sciences, Mashhad, Iran; e Nutrition and Biochemistry Department, School of Medicine, Social Development and Health Promotion Research Center, Gonabad University of Medical Sciences, Gonabad, Iran; f Department of Clinical Biochemistry and Nutrition, Faculty of Medicine, Kurdistan University of Medical Sciences, Sanandaj, Iran; g Student Research Committee, Mashhad University of Medical Sciences, Mashhad, Iran; h Department of Public Health, North Khorasan University of Medical Sciences, Bojnurd, Iran; i International UNESCO Center for Health-Related Basic Sciences and Human Nutrition, Mashhad University of Medical Sciences, Mashhad, Iran

**Keywords:** Inflammation, Oxidative stress, Propolis, Rheumatoid arthritis

## Abstract

**Backgrounds and aims:**

Rheumatoid arthritis (RA), is immune-inflammatory disease which is associated with great pain and disability. Overproduction of pro-inflammatory cytokines and oxidative stress play an important role in RA pathogenesis and related outcomes. The aim of this study was to evaluate the effects of propolis on inflammatory biomarkers and oxidative stress status in RA patients.

**Methods/design:**

Randomized, placebo-controlled, and double-blind clinical trial aiming to recruit 48 patients with RA. Block randomization will be used. An intervention group will receive 500 mg/twice a day propolis capsules for 3 months and control group will receive the placebo for the same dose and duration. The oxidative stress status (malondialdehyde (MDA), total antioxidant capacity (TAC), total oxidant status (TOS), superoxide dismutase (SOD), catalase (CAT), glutathione peroxidase (GPx)), and inflammatory biomarkers (interleukin-17 (IL-17), Tumor necrosis factor alpha (TNF-α), High-sensitivity C-reactive protein (hs-CRP)), lipid profile (total cholesterol (TC), high density lipoprotein (HDL-c), low density lipoprotein (LDL-c), and triglyceride (TG)) and also physical activity, anthropometric indices, clinical and nutritional status will be measured at beginning and end of this study. The primary analysis will be based on theintention-to-treat principle.

**Discussion:**

If this randomized clinical trial shows the reduction in inflammatory cytokines and oxidative stress and improves clinical outcome, it would provide evidence for other clinical trials to evaluate the efficacy of propolis supplementation in RA patients.

## 1 Introduction

Rheumatoid arthritis (RA) is inflammatory disease which firstly targets synovial tissues, lead to considerable pain and disability in patients. The prevalence of RA in adults is about 0.5–1.0% [1,2]. It has a considerable negative effects on the quality of life (QOL), and the ability to do daily activities [3]. The clinical studies have been shown that oxidative stress play an important role in the etiology of RA, also evidences demonstrated the increased level of oxidative stress biomarkers and decreased antioxidants status in RA patients [[4], [5], [6], [7], [8]] The reactive oxygen species (ROS) can cause inflammatory responses by activating nuclear factor kappa-B (NF-kB) in in RA [4]. Therefore, supplementation with antioxidant agents may help to relieve symptoms and improve QOL. Today's pharmacologic agents such as non-steroidal anti-inflammatory drugs, corticosteroids, and disease-modifying anti-rheumatic drugs (DMARDs) are available to alleviate RA symptoms, but these agents have some side effects [9]. Therefore, attention to adjuvant treatments, especially dietary supplements has been increased.

Propolis is a resinous hive product that is made by honeybees [10]. The composition of propolis is very complex and dependent on the plant sources [11,12]. Analysis of propolis samples from different regions has identified at least 300 different compounds. The beneficial effects of propolis are mostly attributed to the phenolic components such as flavonoids (flavonols, flavones, flavonones, dihydroflavonols), terpenes, beta-steroids, aromatic aldehydes, and alcohols [13,14]. Literature showed that propolis has positive effects on atherogenesis, diabetes, cancer, oxidative and inflammatory status [[15], [16], [17], [18], [19], [20]]. Based on the current evidences, antioxidant features of propolis is responsible for its therapeutic benefits [16,19]. It improves the antioxidant status and prevents the production of proinflammatory cytokines by inhibiting NF-kB [[21], [22], [23]].

To the best of our knowledge, there is no clinical trial that assessed the effects of propolis on biochemical and clinical status in RA, so we hypothesized that propolis would decrease serum inflammatory biomarkers, improve oxidative stress status, and clinical status. Hence, this study has been designed to investigate the efficacy of propolis administration in RA patients.

## 2 Methods/design

### 2.1 Trial design

**Fig. 1:**
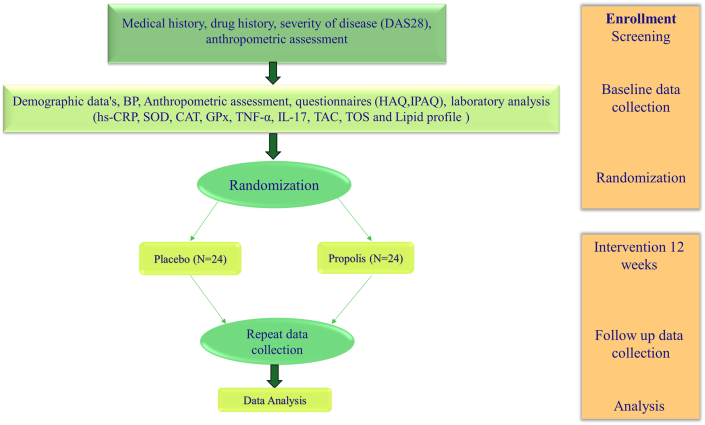
Trial protocol

This RCT protocol was written according to the CONSORT SPIRIT 2013 guidelines [24]. In the present double-blind, randomized, placebo-controlled parallel study we will include 48 women with RA. The study design is presented in Fig. 1. Subjects will be selected from patients referring to the Imam Reza hospital related to Mashhad University of Medical Sciences (MUMS) from Aguste 2020. Patients will be screening based on inclusion and exclusion criteria by an experienced rheumatologist.

### 2.2 Eligibility criteria

Eligibility criteria for RA patients will be done as follows. Participants will be allocated into two groups by block randomization.

### 2.3 Inclusion criteria

- Diagnosis of the disease by a rheumatologist based on the criteria of the American College of Rheumatology [25].
- Women in the age range of 20–70 years.
- Identical drug advisement during study in both of groups.
- Patients with moderate and severe disease activity
- Participants with a history of antioxidant supplements use in three months leading to the study were also excluded
- The tendency to cooperate and sign informed written consent

### 2.4 Exclusion criteria

- Pregnancy and lactation
- Taking oral contraceptive pills
- A history of chronic diseases (i.e. cardiovascular disease (CVD), diabetes, cancer, liver or kidney disease)
- Having other autoimmune and inflammatory diseases
- Abnormal hormonal and thyroid disorders
- Alcohol consumption and hookah smoke
- Smoking and being exposed to secondhand smoke (Active or passive smokers)
- Consumption of any steroidal drugs

### 2.5 Baseline assessment

The study timeline is presented in Table 1. The baseline assessment related clinical status (DAS 28) will be performed by rheumatologist, then the research dietitian, who will collect demographic details, medical and social history (living situation, marital status, current occupation), Health activity questionnaire (HAQ), physical activity questionnaire, dietary intake (3 day record) assessment, anthropometric measurement (Weight, Height, body mass index (BMI), waist circumference (WC) and hip circumference (HC).

**Table 1:**
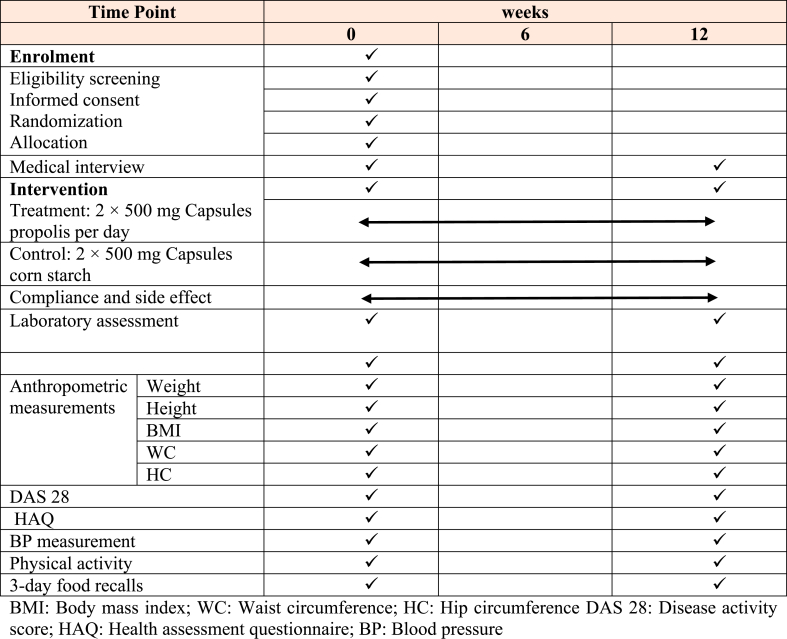
Timeline and applied tests.

### 2.6 Randomization and interventions

Eligibility of patients will be determined at the first screening visit. Block randomization method will be used for this trial study. Participants will be allocated randomly (1:1) to intervention and control groups based on random block procedure consisting of four subjects per block.

Blocking will be complemented according to patient's baseline characteristics such as severity of disease (moderate or severe), menstrual status (yes or no), BMI (BMI: <30 and ≥ 30 kg/m^2^) and type of intervention (propolis or placebo). The intervention group will receive 500 mg propolis (100 mg polyphenol compounds and 67 mg flavonoids) capsules orally twice a day and the control group will receive 500 mg placebo (corn starch) orally twice a day. The drug treatment protocol in the control group will be the same in both of groups. Also, Participants will be instructed to continue their usual diet, physical activity, and medication during the intervention.

Anthropometric indices (Body weight, BMI, WC, HC), and blood pressure will be assessed at beginning and end of intervention. The dietary intakes of patients will be assessed based on 3-day food records and convert to analytical data by Nutritionist IV program modified for Iranian foods. To evaluate the physical activity, we will be used the International Physical Activity Questionnaire (IPAQ) [26].

### 2.7 Ethics approval

The study has been approved by the ethics committee of Mashhad university of Medical Sciences (ethical code: IR. MUMS.MEDICAL.REC.1399.145) and was registered in the Iranian Registry of Clinical Trials website (IRCT ID: IRCT20190407043194N2).

### 2.8 Safety consideration

During the screening and follow-up any abnormalities detected will be discussed with rheumatologist involved in the study. Also, if we see any side effects after consumption of propolis, the patient will be excluded from the study. All participants will be informed of their blood test results as the study will be completed.

### 2.9 Power calculation and sample size estimates

To determine the sample size in this study, we used the previous study [27]. Also, we used from sample size formula suggested for randomized clinical trials. We considered the Type I error of 5% (α = 0.05) and Type II error of 20% (β = 0.2; power = 80%) and serum levels of MDA as a primary outcome, and we calculated the sample size of 18 persons for each group. With 30% dropout, finally twenty-four patients will consider for each group.

### 2.10 Blinding

Patients and researcher team will remain blinded to complete treatment period of 3 months. Treatment allocations will only be revealed at the end of study. The placebo capsule is similar to the study drug for taste, color, size and both of supplements manufacture with identical company (Shahdineh Golha pharmaceutical Company, Isfahan, Iran).

### 2.11 Applied tests during the study

#### 2.11.1 Medical interview

At baseline and after the 3 months intervention, we will assess patients and collect data of the number of tender and swollen joints on the basis of the 28-joint count, Visual Analogue Scale (0–100 mm) for DAS-28 and pain.

### 2.12 Assessment of anthropometric measurements

Weight and height will be measured at the beginning and the end of the study without shoes and minimal clothing by a trained attendant. BMI will be calculated using the height and weight measurements (weight in kg/[squared height in meters]). WC will be measured at the midpoint of the lowest rib and iliac crest.

### 2.13 Laboratory assessment

Seven ml of venous blood will be obtained from all of patients after 10–12 h fasting at the beginning and after 3 months of intervention in the Sadra lab. The serum and plasma will be separated by centrifugation at 3000 rpm for 10 min, then the serum samples will be stored at −70 °C after centrifugation until assay. Finally, Serum hs-CRP, SOD, CAT, GPx, TNF-α, IL-17, TAC, and TOS concentrations will be quantified by the use of a commercial ELISA kit. Spectrophotometric method will be used to determine serum MDA by using the thiobarbituric acid reactive substance method [28]. Serum levels of triglycerides (TG), total cholesterol (TC), and high-density lipoprotein cholesterol (HDL-C) will be measured enzymatically. Low-density lipoprotein cholesterol (LDL-C) will be calculated by the Friedwald formula as follows [29]:

LDL cholesterol = (Total cholesterol-HDL cholesterol) – (Triglyceride/5)

### 2.14 Statistical methods

All our analyses will be based on the intention-to-treat (ITT) approach. Data will be analyzed by using the SPSS software, version 23 (SPSS Inc, Chicago, IL, USA). Quantitative data will be presented as mean ± standard deviation (SD), and qualitative data are will demonstrated as frequency and percent. The Kolmogorov-Smirnov test will be used to assess the normality of data. Paired *t*-test will be used for before and after intervention comparisons. Analysis of covariance (ANCOVA) will be applied to identify any differences between two treatment groups after adjusting for confounding variables. Results will be considered statistically significant at p-value less than 0.05.

### 2.15 Outcome measures

The primary outcome of this trial is the change in serum levels and inflammatory (IL-17, TNF-α, TAC ، TOS, and MDA markers between two groups.

### 2.16 Secondary outcomes include

1 Comparison of DAS 28 score changes in two group after 3 months intervention
2 Comparison of HAQ score changes in two group after 3 months intervention
3 Comparison of Antioxidant enzymes (Superoxide dismutase (SOD), catalase (CAT), Glutathione peroxidase (GPx) changes in two group after 3 months intervention.
4 Comparison of Lipid profile (LDL, HDL, TC, and Triglycerides) mean changes in two group after 3 months intervention

## 3 Discussion

This study is the first clinical trial that will assess the effect of propolis supplementation on inflammation biomarkers and oxidative stress status in women with RA. RA is result the continuous deterioration of cells and tissues [30]. Both oxidative stress and inflammation are considered as main causes in RA pathogenesis [31]. Previous studies have been shown that there is the pro-oxidant/antioxidant imbalance in RA [[32], [33], [34]]. Reducing inflammation and oxidative stress associate with a reduction in the risk of RA. Pro-inflammatory cytokines such as TNF-α and IL-6 play an important role in the pathophysiology of RA [35]. TNF-α and IL-6 are main mediators of cell migration and inflammation in RA [36]. If propolis supplementation reduces inflammation and oxidative stress and improve antioxidant status in this trial, using of propolis as an herbal medicine and nutritional agent can apply for reducing symptom and ameliorating clinical status in RA patients.

Propolis has strong anti-inflammatory function [37]. It able directly and indirectly decrease pro-inflammatory cytokines [38]. Based on the previous clinical trial studies propolis has numerous useful chemical composition with wide phytochemical and antioxidant characteristics that has beneficial effects in human health [16,17,39,40]. Recent meta-analysis that investigated the effect of propolis on inflammatory biomarkers have shown that propolis significantly reduced IL-6, CRP, and TNF-a [41]. Also according to experimental studies propolis prohibit leukotriene and prostaglandin production [42]. The effect of propolis on cyclooxygenase (COX) may be due to flavonoids, which have been shown to suppressed prostaglandin endoperoxide synthase [43]. It also blocks the activation of COX-1, COX-2 [44]. We hope that propolis will improve outcomes by this effect.

## 4 Conclusion

We describe the protocol for a clinical trial design evaluating the effects of propolis supplementation on inflammatory biomarkers, oxidative stress status, and clinical symptoms of RA patients. We expect that oral supplementation of 1000 mg/day of propolis for 3 months, will improve clinical outcomes and decrease the inflammation and oxidative stress in the RA patients. The result of the current study, positive or negative, could provide a step change in the evidence guiding current and future policies regarding the use or not of propolis as complementary treatment in RA patients.

## Trial registration

This trial is registered at clinicaltrials.gov (ID: IRCT20190407043194N2) on July 22, 2020.

## Trial status

The trial enrollment started on 5 Aguste 2020 and currently is recruiting patients. Follow-up and collection labour data of patients expected to take about 6 months.

## Authors’ contributions

ENE, MK, MHJ, MS and MN conceived and designed the study. HT is responsible for statistics analysis. NP and MM collaborated to perform of the clinical trial.

## Funding

This clinical trial is funded by 10.13039/501100004748Mashhad University of Medical Sciences.

## Declaration of competing interest

None declared.

## Abbreviations

**RA**: Rheumatoid Arthritis
ROS: Reactive Oxygen Species
NF-kB: Nuclear Factor Kappa-B
DMARDs: Disease-Modifying Anti-Rheumatic Drugs
DAS 28: Disease Activity Score 28
HAQ: Health Activity Questionnaire
BMI: Body Mass Index
IPAQ: Physical Activity Questionnaire
BP: Blood Pressure
WC: waist Circumference
HC: Hip Circumference
Hs-CRP: High-Sensitivity C - reactive protein
SOD: Superoxide Dismutase
CAT: Catalase
GPx: Glutathione peroxidase
TNF-α: Tumor Necrosis Factor alpha
TAC: Total Antioxidant Capacity
TOS: Total Oxidant Status
MDA: Malondialdehyde
TG: Triglycerides
TC: Total cholesterol
HDL-C: High-density lipoprotein cholesterol
LDL-C: Low-density lipoprotein cholesterol
IL-17: Interleukin 17
