# Supplementary material for: The effect of propolis supplementation on inflammatory factors and oxidative status in women with rheumatoid arthritis: Design and research protocol of a double-blind, randomized controlled
Source: Contemp Clin Trials Commun. 2021 Jun 23;23:100807. doi: 10.1016/j.conctc.2021.100807 (PMC8253959; doi:10.1016/j.conctc.2021.100807)
Supplement: Supplementary file 1 [file mmc1.doc]

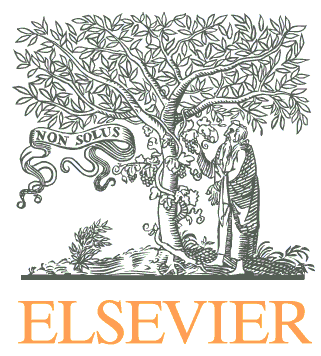


***Contemporary Clinical Trials Communications***

**Conflict of Interest Policy**

**The Effect of Propolis Supplementation on Inflammatory Factors and Oxidative Status in women with Rheumatoid Arthritis: Design and research protocol of a double-blind, randomized controlled**

**Elyas Nattagh-Eshtivani, Mohammadhassan Jokar, Hamed Tabesh, Mohsen Nematy, Mohammad Safarian, Naseh Pahlavani, Mona Maddahi, Maryam Khosravi ⃰**

**Declarations**

***Contemporary Clinical Trials Communications*** requires that all authors sign a declaration of conflicting interests. If you have nothing to declare in any of these categories then this should be stated.

**Conflict of Interest**

A conflicting interest exists when professional judgement concerning a primary interest (such as patient’s welfare or the validity of research) may be influenced by a secondary interest (such as financial gain or personal rivalry). It may arise for the authors when they have financial interest that may influence their interpretation of their results or those of others. Examples of potential conflicts of interest include employment, consultancies, stock ownership, honoraria, paid expert testimony, patent applications/registrations, and grants or other funding.

**Please state any competing interests**

| The authors have no conflict of interest to declare. |
| --- |

**Funding Source**
All sources of funding should also be acknowledged and you should declare any involvement of study sponsors in the study design; collection, analysis and interpretation of data; the writing of the manuscript; the decision to submit the manuscript for publication. If the study sponsors had no such involvement, this should be stated.

**Please state any sources of funding for your research**

| This work was supported by Mashhad University of Medical Sciences |
| --- |

**Signature**

Elyas Nattagh-Eshtivani

Mohammadhassan Jokar

Hamed Tabesh

Mohsen Nematy

Mohammad Safarian

Naseh Pahlavani,

Mona Maddahi,

Maryam Khosravi ⃰
